# Bioinformatic exploration of RiPP biosynthetic gene clusters in lichens

**DOI:** 10.1186/s40694-025-00197-6

**Published:** 2025-05-02

**Authors:** Anna Pasinato, Garima Singh

**Affiliations:** 1 Department of Biology, University of Padova, Via U. Bassi, 58/B, Padova, 35121 Italy; 2 National Biodiversity Future Center (NBFC), Piazza Marina, 61, Palermo, 90133 Italy; 3 Botanical Garden of Padua, University of Padova, Padova, Italy

**Keywords:** Lichenized fungi, Biosynthetic genes, Secondary metabolites, Genome mining, Peptides

## Abstract

**Background:**

Ribosomally synthesized and posttranslationally modified peptides (RiPPs) represent a relatively recent addition to the biosynthetic gene cluster (BGC) repertoire of fungi. These BGCs are primarily involved in toxins production and defense-related functions and resulting metabolites also have a significant therapeutic potential. While only a limited number of fungal RiPPs, primarily from a few model fungi, have been characterized, genome mining approaches show that RiPP BGCs are nearly ubiquitous across the fungal kingdom. However, the RiPP biosynthetic landscape of fungi involved in intricate relationship as symbiosis, such as lichen-forming fungi (LFF), remains unexplored.

**Results:**

This study presents the first comprehensive survey of RiPP BGCs across 111 LFF genomes employing an integrative framework that combines genome mining, phylogenetic inference, and gene network reconstruction. We identified 987 RiPP BGCs, constituting approximately 17% of the total biosynthetic diversity in LFF, a proportion significantly higher than previously estimated. Most lichen RiPP BGCs are unique and do not cluster with any known RiPP gene cluster. We found two RiPP BGCs that were shared among the members of the family Parmeliaceae (Lecanoromycetes), with the signature gene homologous to ustiloxin signature enzyme, indicating a putative similarity to fungal mycotoxin-related BGCs. While one of these BGCs, members of Clan R1, contains the accessory genes for dikaritin synthesis (tyrosinase and methyltransferase), the accessory genes of other BGCs, members of Clan R2, have not yet been reported from any characterized fungal RiPP BGC but only from bacteria. Additionally, for lichen RiPP BGCs that do not cluster with any known BGCs in the RiPP network, we unraveled the presence of the conserved HXXHC motif in the signature gene and, based on this we report the widespread distribution of putative dikaritin homologs across Lecanoromycetes.

**Conclusions:**

This study highlights the presence and distribution of RiPP BGCs in Lecanoromycetes and identifies two conserved RiPP clusters putatively homologous to dikaritins (involved in mycotoxin production) within the Lecanoromycete family Parmeliaceae and a general prevalence of putative signature dikaritin genes (not the cluster) in Lecanoromycetes. Our study highlights the widespread presence of putative mycotoxin-related BGCs in lichenized fungi.

**Supplementary Information:**

The online version contains supplementary material available at 10.1186/s40694-025-00197-6.

## Background

Natural products (NPs) and their derivatives constitute a cornerstone of drug discovery, contributing significantly to the development of new medicines [1]. Among the various classes of secondary metabolites, peptides, specifically the ribosomally synthesized and posttranslationally modified peptides (RiPPs), have emerged as promising candidates for addressing key challenges in drug development, including the modulation of “undruggable” protein–protein interactions and combating antibiotic resistance [2, 3]. RiPPs, owing to their structural diversity and specificity, offer unique therapeutic opportunities by modulating these interactions. In fact, the exploration of fungal RiPPs is timely given the pressing demand for innovative drug leads [4–6]. While RiPPs have been intensively studied in bacteria, fungal RiPPs have only recently been identified. For example, the bacterial RiPP landscape has been well explored, and some bacterial RiPPs are already utilized as medicines, such as the lanthipeptide duramycin for cystic fibrosis [7, 8] and the thiopeptide LFF571 for *Clostridium difficile* infections [9]. On the other hand, research on fungal RiPP BGCs (biosynthetic gene clusters) remains relatively recent and has focused mainly on fungal classes comprising model fungi such as Eurotiomycetes, Basidiomycetes, and Saccharomycetes. However, the distribution patterns and diversity of RiPP-related BGCs in fungi engaging in intricate symbiotic associations, such as lichens, remains largely unexplored. This gap may be attributed to the difficulty of cultivating them in axenic conditions and the slow progress of genome sequencing in this group, which has only recently gained attention.

To date, six classes of fungal RiPPs have been identified, including amatoxins, phallotoxins, and borosins from Basidiomycota, and dikaritins (encompassing ustiloxins, phomopsins, and asperipins) and epichloëcyclins from Ascomycota [10]. Among these, Basidiomycota RiPPs have been the well studied, primarily due to their toxic effects on humans and their immunosuppressive properties [11, 12]. In contrast, Ascomycota RiPPs remain largely understudied, with research efforts predominantly centered on RiPPs secreted by model fungi in the classes Sordariomycetes and Eurotiomycetes. Most well-studied Ascomycota RiPPs are dikaritins, which are primarily mycotoxins known for their antimitotic activity [13, 14]. Considering that the primary role of RiPPs include antimicrobial, particularly antifungal, and antifeedant activities and that common saprobic fungi are notably absent from lichen thalli, we hypothesize that lichens are likely to be rich reservoirs of RiPP BGCs.

Recent advances in omics technologies and computational tools have revolutionized the discovery of natural products, including RiPPs. Tools such as AntiSMASH, which integrate both known RiPP precursors from the MIBiG 3.1 database and predicted precursors from its internal database, have greatly enhanced the identification of fungal RiPPs in the genomes [15]. In addition to the identification, automated pipelines facilitate comprehensive comparison and clustering of BGCs into gene cluster families, enabling the discovery of both novel and previously characterised RiPP pathways in target organisms as well as pathways shared among organisms [16]. These innovations are critical for facilitating dereplication and identifying the most promising candidates for further investigation, addressing a key challenge in natural product discovery.

In this study, we systematically mine, compare, and quantify the diversity of RiPP BGCs across 102 Lecanoromycetes genomes via an integrative approach that combines phylogenetics, conserved domain identification, and sequence similarity networks. This work represents the first comprehensive analysis of Lecanoromycetes RiPPs, which include 20 taxonomic families and 80 lichenized fungal genera inhabiting diverse ecological and geographical regions. Specifically, we aim to i) identify the diversity of RiPPs within Lecanoromycetes via genome mining, ii) identify the widespread RiPP pathway via gene network analysis, if any, and evaluate the homology of Lecanoromycetes RiPP genes and clusters with those from Eurotiomycetes and Sordariomycetes, and iii) identify potential novel RiPP classes within Ascomycota.

## Materials and methods

### Dataset and phylogenomic analysis

A total of 111 lichenized fungal genomes were included in the study, comprising 102 lichenized fungi belonging to Lecanoromycetes, including lichenized fungi belonging to Dothideomycetes and Eurotiomycetes as outgroups, to contextualize our findings. All the genomes are publicly available at NCBI (https://www.ncbi.nlm.nih.gov/) or JGI portal Mycocosm (https://mycocosm.jgi.doe.gov/mycocosm/home). Genome completeness was estimated via BUSCO v.5.3.2 and using ascomycetes_odb10 dataset [17] (Supplementary material S1). To construct the phylogenomic tree, universal single-copy genes were compared to filter out those present in most taxa (a maximum of one sample missing). The single-copy BUSCO genes were then concatenated, and the concatenated sequences from all the taxa were aligned via MAFFT L-INS-I [18]. Phylogenetic relationships were inferred from the alignment by implementing maximum likelihood (ML) analysis as implemented in IQ-TREE v.1.5.5 [19] using standard model selection and 1,000 bootstrap replicates. The resulting tree was visualized using FigTree v.1.3.1 and annotated using iTOL.

### RiPP identification and clustering using automated genome mining software

The secondary metabolite gene clusters were predicted via the antiSMASH v7.0 fungal version [15]. The program was run with setting the strictness to ‘relax’ and enabling all functions. As some of the genomes were fragmented (> 1,000 scaffolds), which could inflate the number of RiPPs identified, we tested the correlation between the number of scaffolds and the number of BGCs. Pearson’s product‒moment correlation was calculated via the stats v3.6.2 R package. A correlation coefficient close to 0 suggests no correlation between the variables, whereas a value near 1 indicates a strong positive correlation. A correlation coefficient of 0.14 was found suggesting that level of fragmentation doesn’t inflate the number of BGCs identified (Supplementary material S2).

Biosynthetic gene similarity clustering and prospecting engine (BiG-SCAPE) [20], a platform to compare and group similar BGCs into gene cluster families (GCFs) on the basis of distance metrics, was used to quantify BGC diversity. BiG-SCAPE constructs sequence networks of BGCs based on their protein domain architecture and sequence identity. BGCs similar in gene sequence and content are grouped into GCFs, and two or more syntenic or homologous GCFs can be further clustered into clans potentially coding for similar compounds.

BGCs identified by AntiSMASH were compared against the MIBiG database, which comprises characterized fungal RiPPs by implimenting the BiG-SCAPE pipeline and using the “-mibig” flag [21]. We computed the BGC assignment into GCFs via a conservative approach‒the raw distance cutoff of 0.6‒to avoid overestimating the number of potentially novel BGCs. The analysis was performed with the default settings in the ‘auto’ mode, with singletons retained, and with the PFAM database.

Clinker was used to compare and visualize the synteny and homology of RiPP gene cluster clans [22]. We then inferred the RiPP diversity associated with different families within Lecanoromycetes to identify any putative RiPP diversity hotspots among taxa.

### Bioinformatic characterization of RiPP gene clusters

To better understand the genes involved in the synthesis and modification of RiPPs, it is essential to identify conserved accessory genes in BGCs; therefore, we performed a conserved domain search on each accessory gene part of the clan BGCs and annotated the results in clinker [22].

AntiSMASH identified the signature enzymes of the two clans as UstY homologs. To confirm the class-defining motifs and conserved amino acids of the signature enzymes in the two RiPP clans, the signature biosynthetic genes were aligned with the UstYa and UstYb protein sequences from *Aspergillus flavus* (GenBank accessions: QRD84928.1 and QRD84930.1) as references using the alignment program MUSCLE (Supplementary material S3). Phylogenetic relationships were inferred from the alignment via ML analysis as implemented in IQ-TREE v2.3.2 via standard model selection and 1,000 bootstrap replicates. The resulting tree was visualized and annotated in iTOL. The alignment of the most conserved region was cut and visualized via WebLogo [23].

### Identification of the precursor peptide

Although antiSMASH v7.0 can identify precursor peptides present in the MIBiG 3.1 database, these precursors were not identified in the two RiPP clans, probably due to the high variability of these sequences. The presence of UstY homologs in both clans suggests that these homologs could have a ustiloxin-like precursor peptide [24].

We therefore identified all signal peptides in the RiPP BGCs that constitute the clan, using the SignalP6.0 algorithm [25] and then tested the identified sequences for the presence of Kex2 cleavage sites via a custom pipeline (Supplementary material S4). Finally, we identified tandem repeats using the radar tool from EMBL (https://www.ebi.ac.uk/jdispatcher/pfa/radar).

### Putative dikaritin homologous sequence analysis

While Lecanoromycetes RiPP BGCs did not show strong homology with known BGCs present in the MIBiG repository, we checked for the presence of conserved domains in the signature genes. AntiSMASH ClusterCompare algorithm revealed clusters similar to those of asperipin-2a, ustiloxin and phomopsin, with similarity scores varying from 0.1 to 0.3. ClusterCompare compares gene clusters considering presence of biosynthetic profiles, gene functions, and sequence identity and synteny. Amino acid sequences of the signature enzyme belonging to the asperipin-2a, ustiloxin and phomopsin identified gene clusters were aligned in Geneious Prime via the MUSCLE algorithm. The presence or absence of each dikaritin homolog across the taxa was then annotated via iTOL in the phylogenomic tree.

### Phylogenetic analysis of clan R1 methyltransferases

To understand the putative function of the conserved methyltransferases found in Clan R1 BGCs, all O-methyltransferases and SAM-dependent methyltransferases of the clan were extracted and aligned with the methyltransferases identified in various fungal dikaritin BGCs [13] using MUSCLE (Supplementary material S5). The ML tree was generated with IQ-TREE v2.3.2 using standard model selection and 1,000 bootstrap replicates and visualized in iTOL.

## Results

### RiPP BGC distribution and diversity in lichenized fungi

**Fig. 1:**
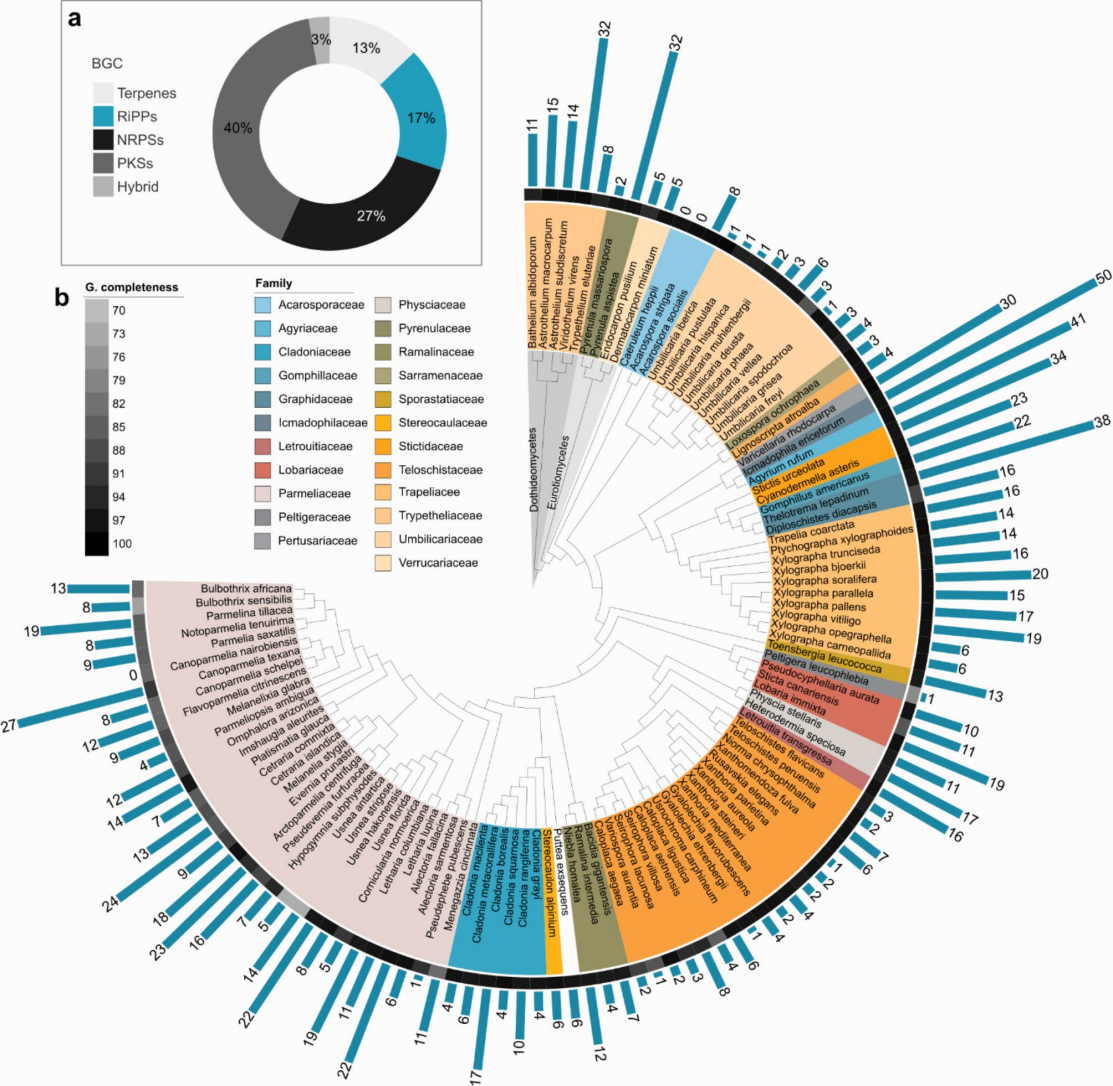
Overview of total biosynthetic gene clusters (BGCs) and distribution of RiPPs in the selected species. **(a)** Relative abundance of the 6,642 BGCs identified in our dataset. RiPPs constitute 17% of the total BGCs. Other major BGC classes detected are - PKSs: Polyketide synthases; NRPSs: nonribosomal peptide synthetases; terpenes and Hybrid: hybrid chemical products of nonribosomal peptides and polyketide synthases. **(b)** A phylogenetic tree inferred from universal single-copy genes showing the distribution of RiPP biosynthetic gene clusters across taxa, depicted as blue bars. Genome completeness (G. completeness) is shown using a heatmap. The number of RiPP BGCs varies greatly among lichen-forming fungi.

AntiSMASH predicted the presence of 1,062 RiPP BGCs in Lecanoromycetes (102 taxa), 59 in Eurotiomycetes (4 taxa), and 79 in Dothideomycetes (5 taxa). RiPP BGCs constitute approximately 17% of the total biosynthetic diversity of lichenized fungi (Fig. 1a). RiPP BGCs are unevenly distributed among lichens, with some taxa, such as *Caeruleum heppi* and *Canoparmelia naerobiensis*, lacking these gene clusters entirely, while others, such as *Icmadophila ericetorum*, harbor a notably extensive repertoire -up to 50 RiPP BGCs (Fig. 1b).

### Lichens have unique RiPP biosynthetic gene clusters

**Fig. 2:**
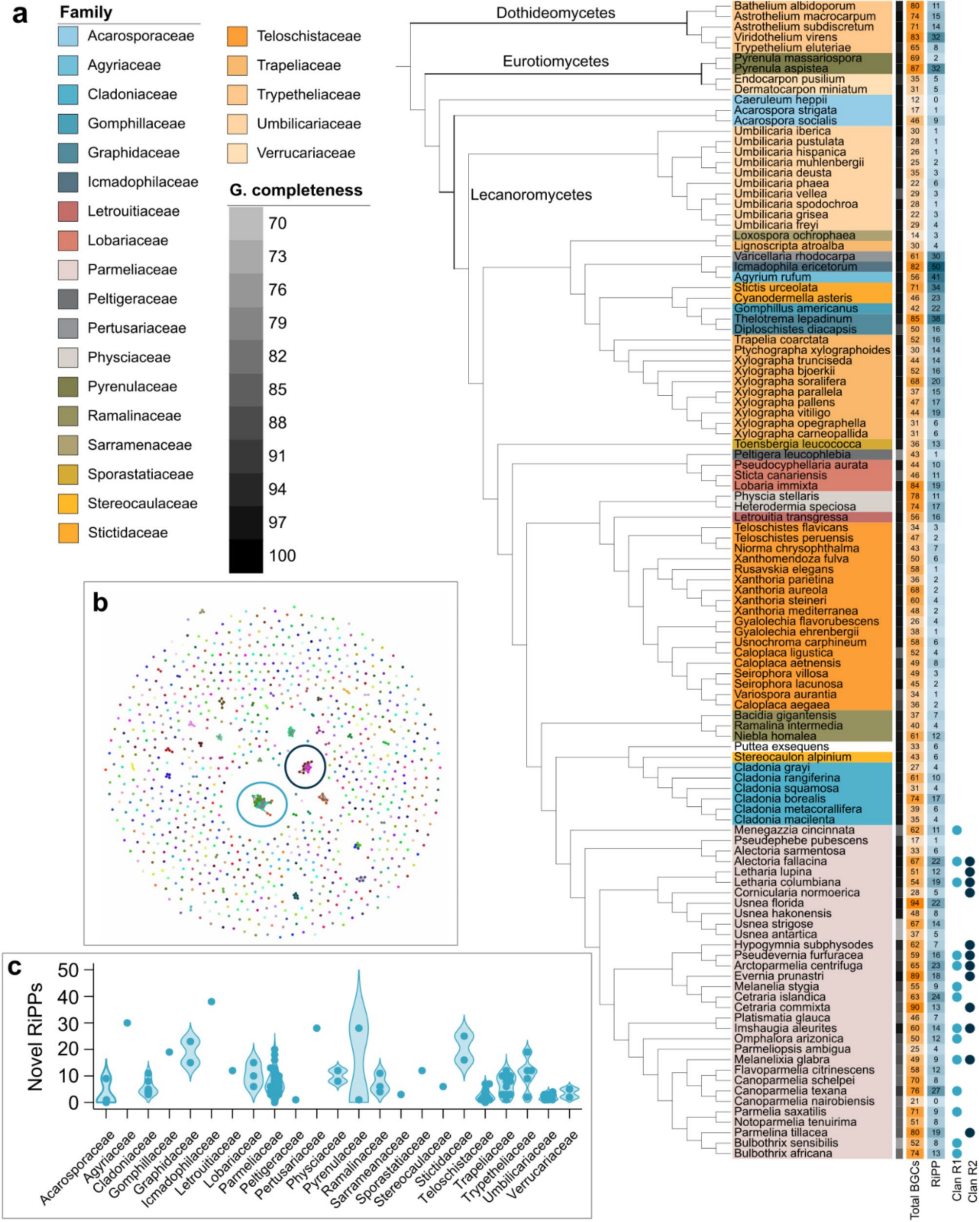
Diversity of RiPP biosynthetic gene clusters (BGCs) and distribution of the conserved RiPP BGCs in lichens. **(a)** Phylogenomic species tree depicting genome completeness (G. completeness; inferred as percentage of complete BUSCO genes present in the genomes), total BGCs and RiPP BGCs identified in each taxon (shown as heatmaps). Blue circles/dots in front of the species name on the tree represent the presence of a BGC belonging to the two conserved RiPP clans. Both RiPP clans were detected in the Parmeliaceae family. **(b)** BiG-SCAPE network of RiPP BGCs. Most RiPP gene clusters do not cluster into GCFs and are represented by single dots in this RiPP network. Some GCFs cluster together, thus forming larger clusters or clans, marked by the colored circles in the network. Clan R1 and Clan R2 are marked by the circles in light blue and dark blue respectively. **(c)** Violin plot demonstrating the distribution of unique RiPP BGCs across taxa in the different Lecanoromycete families.

To identify and characterize homologous RiPP gene clusters, a gene network was constructed which grouped these BGCs into gene cluster families (GCFs) using the BiG-SCAPE program. Among the 987 RiPP BGCs, 827 (~ 95%) were singletons, as they did not cluster with any other BGCs, potentially coding for unique peptides. The remaining 160 RiPP BGCs (~ 5%) clustered into 48 GCFs (Fig. 2b). All lichenized fungal families presented unique RiPP gene clusters, with Icmadophilaceae and Agyriaceae being the most diverse (Fig. 2c). The gene cluster network revealed the presence of two clans, formed by different GCFs, in the Parmeliaceae family (Fig. 2a and c). To understand the evolution of these conserved RiPP gene clusters, the two RiPP BGC clans—Clan R1 and Clan R2—were examined more closely via cluster synteny analysis. In summary, RiPP BGCs were grouped into GCFs based on predicted similarity, with further hierarchical clustering revealing two larger clans within the Parmeliaceae family, which were analyzed in detail to explore their evolutionary patterns.

### Evolutionary conservation of clans: cluster synteny

We found that most lichen RiPP BGCs are unique and species-specific. However, two homologous clans restricted to Parmeliaceae were identified, potentially coding for similar compounds. The two RiPP clans share a signature gene with a domain of unknown function (DUF3328), which is found in oxidases homologous to UstYa and UstYb and is involved in dikaritin biosynthesis.

**Fig. 3:**
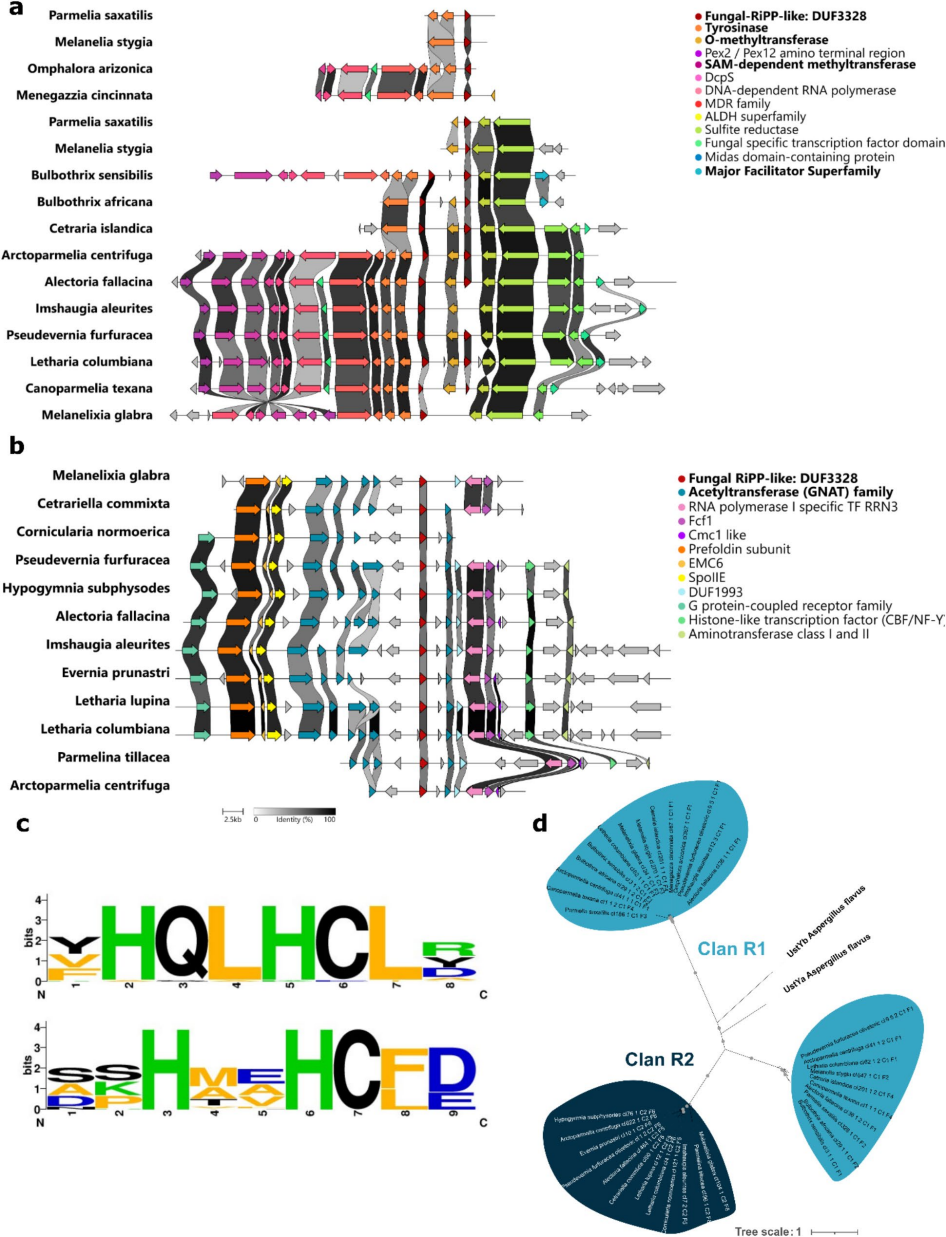
Cluster synteny of the two RiPP clans. **(a)** Cluster synteny of Clan R1. Enzymes putatively involved in RiPP maturation/transport are in bold. **(b)** Cluster synteny of Clan R2. Enzymes putatively involved in RiPP maturation/transport are in bold. **(c)** Sequence logo of the two conserved HXXHC motifs found in the signature genes of the clans, hypothesized to be the active site of UstY homologs. **(d)** Gene tree depicting the relative distance between the signature genes in the two clans. Bootstrap support (> 70%) is indicated by gray dots

Clan R1 typically contains two signature biosynthetic genes, with taxa such as *Parmelia saxatilis* and *Melanelia stygia* presenting two BGCs, each containing one signature gene. These taxa likely have one gene cluster that is fragmented because of incomplete genome assembly. The conserved accessory genes in Clan R1 include tyrosinase, O-methyltransferase, and FGE-sulfatase, with tyrosinase located upstream of the signature gene and O-methyltransferase (OMT) located between the two signature genes, suggesting their roles in product maturation. Fewer conserved domains are involved in transcription regulation and transport (Fig. 3a). Methyltransferases identified in Clan R1 BGCs form two distinct clades: OMTs and SAM-dependent methyltransferases. SAM-dependent methyltransferases exhibit closer evolutionary relationships to methyltransferases identified in the dikaritin BGCs of *Metarhizium* and *Ophiocordyceps*, whereas Clan R1 OMTs form a distinct clade (Supplementary material S6).

Clan R2 contains multiple copies of an acetyltransferase from the GNAT family, with two conserved copies flanking the signature gene. Clan R2 also includes less conserved enzymes involved in posttranslational modifications, such as cytochrome c oxidase biogenesis proteins, aminotransferases, and chaperone proteins such as prefoldin, along with a domain of unknown function (DUF1993) (Fig. 3b).

### Signature biosynthetic gene conservation of RiPP clans

Although the signature genes of both clans contain a region coding for DUF3328, which is characteristic of UstY homologs, BGCs from neither clan clustered with fungal dikaritin BGCs present in the MIBiG repository. To further investigate the evolutionary conservation of the signature genes in these clans, the sequences were aligned using Geneious Prime. The alignment revealed overall low sequence conservation, except for two highly conserved HXXHC motifs (Fig. 3c). Phylogenetic analysis of the signature genes revealed greater sequence similarity within each clan than between clans. Notably, the signature biosynthetic genes of Clan R1 were split into two distinct phylogenetic clades (Fig. 3d).

Interestingly, genes putatively containing signal peptides for protein translocation to the endoplasmic reticulum were identified in some RiPP BGCs, as were putative recognition sites for the Kex2 protease localized in the Golgi apparatus. These features are essential for the maturation of ustiloxin-like peptides and suggest the presence of a gene encoding the precursor peptide. However, these genes lack conserved repeated motifs, one of the signature features of dikaritin core peptides.

### Dikaritin homologs in Lecanoromycetes

To identify the closest characterized homologs of the lichen RiPP BGCs that did not group with the Clan1 or Clan2 in BiGSCAPE, we examined the conserved domains in each RiPP signature gene detected by AntiSMASH. The ClusterCompare algorithm integrated within AntiSMASH pipeline revealed some sequence similarity to the asperipin-2a, ustiloxin and phomopsin signature genes, with similarity scores varying from 0.1 to 0.3. These BGCs did not cluster as a clan in the RiPP network generated by BiG-SCAPE probably because of the low sequence similarity and low cluster homology. Alignment of the signature genes of asperipin-2a, ustiloxin, and phomopsin homologs revealed the presence of the same conserved motif (HXXHC), confirming their relationship with dikaritins despite the low similarity.

**Fig. 4:**
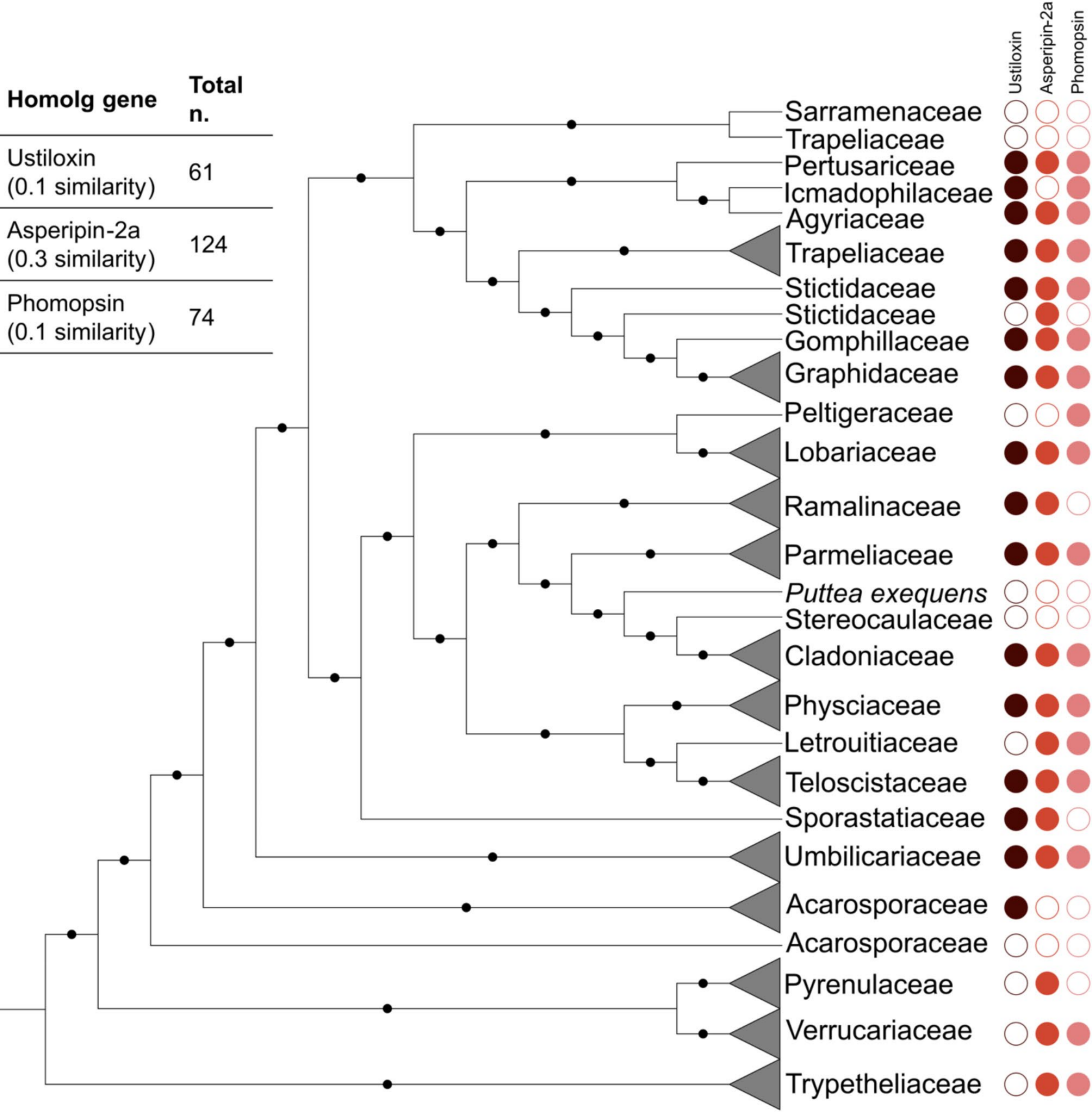
To putatively identify the remaining lichen RiPP BGCs (those not in BiGSCAPE clans) with their closest known homologous cluster, we examined the conserved domains in each RiPP signature gene detected by AntiSMASH. Based on the presence or absence of these domains, we classified the lichen RiPPs into putative ustiloxin, asperipin-2a, and phomopsin homologous gene clusters. It is important to note that this classification is based solely on the conserved domains, not on cluster homology or synteny, meaning these clusters are the closest known homologs but remain distinct from the ustiloxin, asperipin-2a, and phomopsin clusters. This figure shows the distribution of the three signature domains, or their absence, across various lichens. The absence of the domain is indicated by empty circles, presence by colored circles. The three shades indicate three different putative dikaritin gene clusters identified by MIBiG integrated within AntiSMASH (similarity ranging from 0.1 to 0.3). Dark brown circles represents putative ustiloxin homologs, red ones indicates asperipin-2a, and pink ones denotes phomopsin homologous gene clusters. These clusters are distinct from those that cluster within R1 and R2 clans. The black dots indicate bootstrap values > 70.

The phylogenetic distribution of these putative dikaritin homologs is depicted in Fig. 4. All putative dikaritin homologs are present in different Lecanoromycete families.

## Discussion

### Lichenized fungi are a treasure chest of novel RiPPs

This is the first study shedding light on the diversity of lichenized fungal RiPP BGCs. We found that RiPP BGCs constitute approximately 17% of the total BGCs in lichenized fungi, as opposed to the 0.5% suggested for fungi, including lichen-forming fungi [26]. However, AntiSMASH predicts RiPP BGCs based on the presence of a peptidase alongside a tetrapyrrole methylase gene, a cytochrome P450 gene or a DUF3328 protein-encoding gene. Although this approach may lead to the exclusion of RiPP BGCs with divergent gene compositions, for example, those lacking a peptidase, such as borosins, asperipin-2a and the ustiloxin gene cluster in *Ustilaginoidea virens*, it represents a significant advance in RiPP detection compared to the previously available pipelines. The RiPP BGCs of fungi have been shown to be very divergent in both gene sequence and cluster composition, making the general guidelines for their identification and classification difficult [16, 27]. Nonetheless, these pipelines already demonstrate that RiPPs constitute a significant portion of LFF BGCs, and this proportion is likely to increase with the discovery of additional RiPPs and the subsequent refinement and expansion of BGC detection pipelines.

Lichenized fungi are known to predominantly produce species-specific or mostly taxonomically restricted secondary metabolites, as highlighted in several studies [28–35], with the exception of some PKS derivatives. We observed a similar trend for the diversity of RiPP BGCs in lichenized fungi: most RiPP BGCs appeared as singletons in the network analysis, suggesting that they may encode structurally and functionally unique compounds (Fig. 2b, c).

RiPP BGCs in non-lichenized model fungi have been shown to encode toxins [36]. The low sequence homology and overall conservation of lichen RiPP BGCs may result from finely tuned, species-specific requirements driven by the biotic interactions surrounding the lichen. Toxins, in general, are shaped by long-term evolutionary processes finely tuned to address specific biotic interactions. For example, amatoxins evolved in *Amanita* sp. to counter mycophagy are highly toxic to insects, nematodes, and mammals, including humans [10, 36]. Phallotoxins, on the other hand, although structurally similar to amatoxins, exemplified by phallacidin, have poor absorption in the gut and therefore are not as toxic, and therefore show significant potential as candidates for novel therapeutics [10, 37]. The diversity of RiPP-BGCs discovered in lichenized fungi could therefore reflect species-specific requirements.

### Towards the identification of novel mycotoxin gene clusters

Although the vast majority of lichen RiPP BGCs are unique, two conserved clusters (referred to as Clan R1 and Clan R2), putatively encoding similar compounds, were found in the largest lichenized fungal family, Parmeliaceae (Fig. 2a).

These two RiPP clans (Clan R1 and Clan R2) did not cluster with any other known fungal RiPP BGC and therefore putatively encode novel products. In-depth analysis of the signature biosynthetic genes of the two clans revealed evolutionary closeness to *ustYa* and *ustYb*, genes encoding DUF3328-containing proteins involved in the posttranslational modifications of the precursor peptide. UstY homologs are involved in the oxidative cyclization process of the precursor peptide, as indicated by gene knockout and heterologous expression in *Aspergillus oryzae* [38]. These proteins constitute the class defining enzymes of the dikaritins, predominantly mycotoxins [39]. Despite overall low sequence similarity, two conserved motifs, HXXHC, were found in all the signature genes (Fig. 3c). This conserved double HXXHC motif is suspected to be part of the DUF3328 active site [27, 40]. The presence of these motifs in the signature genes indicates their involvement in RiPP production.

### Clan R1 BGCs are similar to fungal ustiloxin BGCs

The Clan R1 BGCs contain several conserved genes encoding enzymes that are involved in the synthesis of ustiloxin B in *Aspergillus flavus*. For example, the cluster includes two genes encoding DUF3328-containing proteins homologous to UstY and a tyrosinase domain-containing protein located nearby. The combined activity of tyrosinase, UstYa, and UstYb has been reported to introduce the cyclic structure of N-desmethyl ustiloxin F, the first intermediate in the ustiloxin B biosynthetic pathway. Next, an N-methyltransferase (NMT) methylates the amino group of ustiloxin F [38, 41]. A more detailed biosynthetic pathway of ustiloxin biosynthesis can be found in Montalbán-López et al. (2021). Interestingly, both a SAM-dependent methyltransferase (SAM-MT) and an O-methyltransferase (OMT) were identified in Clan R1 BGCs and could play different roles in the maturation of the RiPP. Clan R1 SAM-MTs exhibit closer evolutionary relationships with *Metarhizium* and *Ophiocordyceps* (both Sordariomycetes fungi), despite the significant phylogenetic distance between these taxa and *Aspergillus* RiPP NMTs and *Ustilaginoidea* NMTs. Nonetheless, the methyltransferases involved in dikaritin biosynthesis exhibit functional convergence, despite notable sequence divergence; NMTs from both clades are able to catalyze the conversion of phomopsin A to phomopsin E [13]. O-methyltransferases, on the other hand, catalyze the methylation of an oxygen atom– often in hydroxyl groups– and are involved in modifying small molecules. Although they have never been reported in dikaritin BGCs, they play a key role in amino acid modification in bacterial RiPPs, such as lanthipeptides and lasso peptides [42–44]. These findings suggest that lichen OMTs in Clan R1 could play an active role in RiPP biosynthesis in LFF.

The signature genes of Clan R1 formed two monophyletic clades in the gene tree (Fig. 3a and 3d), one grouping with UstYa and the other with UstYb from *Aspergillus flavus*. This phylogenetic separation suggests that the two signature genes in Clan R1 likely encode distinct products that are homologous to UstYa and UstYb. The final product, ustiloxin B, in *A. flavus* involves many other tailoring enzymes: a cytochrome P450, two flavin-containing monooxygenases, and a PLP-dependent protein, which are absent in Clan R1. This finding suggests that the products of Clan R1 BGCs may not be chemically identical but may still be similar to the known ustiloxin B.

### A novel RiPP BGC? Clan R2 presents unprecedented cluster architecture

Clan R2 contains only a single signature biosynthetic gene homologous to *ustY* (Fig. 3b and c). However, none of the posttranslational modification enzymes characteristic of ustiloxin or other dikaritin biosynthetic pathways are present within its gene cluster [39]. Interestingly, it is not uncommon to have only the signature genes and the absence of tyrosinase or even peptidase in the RiPP BGC. Asperipin-2a, a fungal dikaritin RiPP BGC, for example, contains only a single *ustY*-homologous gene. This unique cluster architecture suggests a potential relationship with dikaritins, possibly asperipin-2a, due to the presence of an *ustY* homolog. Our study revealed that RiPP BGCs not following the same gene architectural logic may be more common and more widespread than previously thought. The BGCs belonging to Clan R2 RiPPs in LFF, however, may be more diverse than asperipin-2a, as the accessory enzymes of this BGC involved in asperipin-2a maturation, a multidrug transporter and a reductase [45], are absent in Clan R2 BGCs.

Interestingly, Clan R2 BGCs contain many copies of an acetyltransferase gene belonging to the GNAT family (Fig. 3b). These enzymes are reported from bacterial RiPPs, such as the linear azoline-containing peptide goadsporin [46], the lasso peptide albusnodin [47], and many microviridins [48]. In all these cases, the enzyme catalyzes the N-terminal acetylation of the core peptide following the removal of the leader peptide. Although the cluster architecture resembles that of fungal asperipin-2a, the entirely distinct set of accessory genes, featuring an acetyltransferase in place of a reductase, indicates that these BGCs constitute a different class of RiPPs.

### The mystery of the precursor peptide: defining a dikaritin BGC

RiPP precursor peptides are highly variable because of a key feature of RiPP biosynthesis -the presence of a recognition site (leader or follower) adjacent to the core peptide, which enhances the promiscuity of tailoring enzymes without constraining the evolution of the core [2, 39]. This high variability could render AntiSMASH unable to identify any putative precursor peptide in the two clans.

Dikaritin precursor peptides typically contain multiple core peptides [39, 49] that exhibit two distinctive features: a signal peptide for translocation into the endoplasmic reticulum and highly repeated core sequences [41]. Precursor peptides are usually positioned an average of three genes away from DUF3328 domain-containing protein genes [24]. Interestingly, no conserved repeated regions were identified in the BGCs of the two RiPP clans studied. The absence of these regions might suggest that the precursor peptide is not located in the BGC or represents a case of a single core peptide.

DUF3328 proteins are tailoring enzymes found in various BGCs, including ustiloxins, phomopsins, asperipin-2a, and epichloëcyclins, where they catalyze diverse modifications. They are known to form ether bonds in ustiloxins, perform hydroxylation and chlorination in cyclochlorotine BGCs, and participate in transacylation reactions. Importantly, although Montalbán-López et al., in their 2021 review, identified DUF3328 protein genes as the defining feature of dikaritins, these enzymes are not exclusive to RiPP pathways, as they are also found in BGCs for polyketides such as atpenin A4 and nonribosomal peptides such as astins, indicating a broad functional role [27, 50, 51].

### Widespread presence of dikaritin homologous genes in lecanoromycetes?

The definition of a dikaritin BGC has been continually refined and expanded with the ongoing discovery of novel fungal RiPPs. While Ding et al. (2015) proposed that dikaritin BGCs are identified by the presence of the following five protein-coding genes: [1] a precursor peptide containing an N-terminal leader peptide and repeated core/recognition motifs [2], a tyrosinase [3], a methyltransferase [4], multiple DUF3328 proteins, and [5] transporter proteins or peptidases; functional RiPP BGCs lacking one or more of these enzymes were reported later. For example, the asperipin-2a and ustiloxin gene clusters in *Ustilaginoidea virens* lack a peptidase gene [39, 52]. Recently, Montalbán-López (2021) redefined this definition and suggested the DUF3328 protein as the class-defining enzyme of dikaritins. Given the diverse natures and functions of these enzymes, the presence of two “HXXHC” motifs was suggested to be a reliable way to detect RiPP-related DUF3328 proteins [27].

We aimed to identify putative RiPP BGCs in lichens that appeared as singletons in the BiGSCAPE network analysis. Examination of the conserved domains in the RiPP signature genes of these clusters revealed the presence of HXXHC motifs in DUF3328 genes, the class-defining enzymes of dikaritins. Further analysis grouped these BGCs with known fungal dikaritins, specifically ustiloxin, asperipin-2a, and phomopsin RiPPs. However, while the closest known homolog of lichen RiPPs belongs to these above-mentioned clusters, it is important to note that this classification is based solely on conserved domains, as the overall cluster homology and synteny among the two differs significantly. This could explain why these clusters were not clustered with the Clan R1 which comprises lichen RiPPs putatively homologous to fungal ustiloxin BGCs in terms of presence of the HXXHC motifs in DUF3328 genes and the overall cluster synteny.

Dikaritins have been identified in Sordariomycetes (ustiloxin from *Ustilaginoidea virens* and phomopsin from *Phomopsis leptostromiformis*), Eurotiomycetes (ustiloxin from *Aspergillus flavus*), and Dothideomycetes (victorin *from Cochliobolus victoriae*) [36]. DUF3328 domain-containing genes have also been identified in a broad range of Ascomycota [24], but they have not yet been reported in lichenized fungi. This study expands the current knowledge on the distribution of putative dikaritin homologs to Lecanoromycetes. Interestingly, the presence of dikaritin homologs is not limited to Parmeliaceae; instead, these fungal RiPPs are spread throughout Lecanoromycetes, Dothideomycetes and Eurotiomycetes (Fig. 4).

While no RiPPs have ever been characterized from Lecanoromycetes, our findings show that lichens are potentially a rich source of RiPPs, especially those related to dikaritins. Notably, these homologs did not cluster together in BiG-SCAPE, suggesting that they possess distinct BGC compositions. This variability indicates that lichenized fungi may produce a wide range of RiPP natural products, representing promising sources of novel bioactive compounds.

## Conclusion

This study represents the first investigation of the diversity of lichenized fungal RiPPs. Our findings revealed that lichen RiPP BGCs differ significantly from those identified in model fungi, suggesting their potential to encode novel compounds. Among these, we identified two conserved RiPP BGCs distributed within the Parmeliaceae family. These BGCs not only contain divergent signature biosynthetic genes from one another but also differ in cluster composition and architecture, suggesting that they could synthesize structurally diverse RiPPs. Furthermore, the signature biosynthetic gene in both BGCs (clans) contains a characteristic domain associated with *ustY* homologs, linking them to fungal dikaritins, a class predominantly composed of mycotoxins. However, the absence of the typical repeated core peptide in these clusters points to the uniqueness of their putative RiPP products. Finally, we extended the known phylogenetic distribution of dikaritin-like genes to include Lecanoromycetes, broadening their evolutionary and functional significance. These findings provide a foundation for advancing our understanding of the biochemical potential underlying the lichen metabolome. Investigating natural products and their biosynthetic machinery in non-model organisms, such as lichens, is crucial for the development of novel therapeutics, with RiPPs offering uniquely versatile scaffolds for drug discovery. While genome mining has revolutionized natural product research, experimental validation of the identified RiPP BGCs—including structure elucidation and bioactivity assays—is a critical next step for utilising these compounds for medical applications.

## Electronic supplementary material

Below is the link to the electronic supplementary material.

Supplementary material S1: Voucher information of the taxa and genomes used in this study

Supplementary material S2: Pearson’s correlation coefficient between the number of scaffolds and the number of biosynthetic gene clusters in the dataset

Supplementary material S3: Sequences of the signature RiPP genes belonging to Clan1 and Clan2

Supplementary material S4: Signal peptides and Kex2 cleavage sites identified in Clans R1 and R2

Supplementary material S5: Sequences of the methyltransferases used to infer the tree are presented in Supplemental material S6. Gene tree of the methyltransferases identified in Clan R1 and reference from Ding et al. (2016). The lichen methyltransferase genes identified in this study are marked in bold

## Data Availability

No datasets were generated or analysed during the current study.
