# Supplementary figures and images for: Bioinformatic exploration of RiPP biosynthetic gene clusters in lichens
Source: Fungal Biol Biotechnol. 2025 May 2;12:6. doi: 10.1186/s40694-025-00197-6 (PMC12048977; doi:10.1186/s40694-025-00197-6)

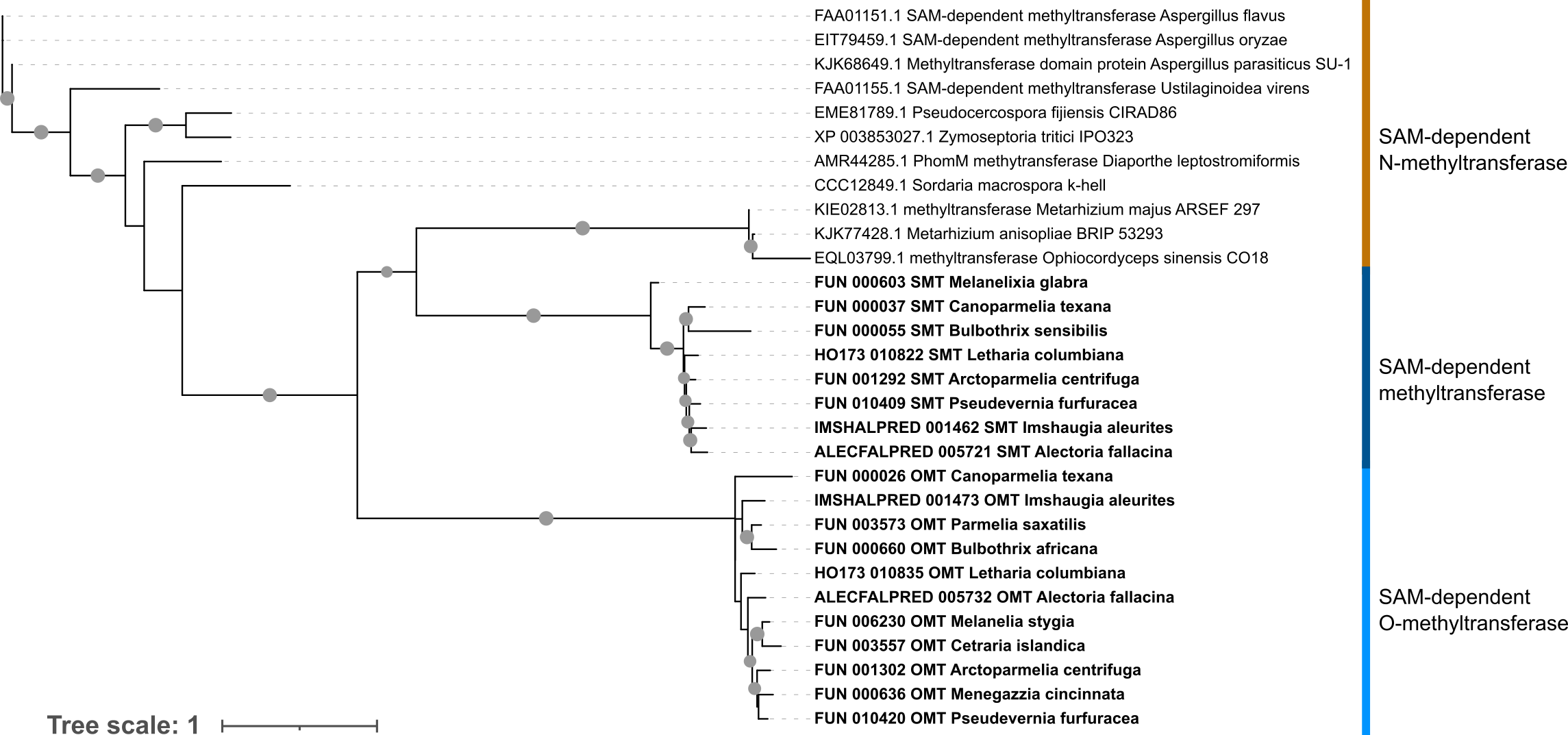

Supplement: Supplementary file 3 — Supplementary material S3: Sequences of the signature RiPP genes belonging to Clan1 and Clan2 [file 40694_2025_197_MOESM3_ESM.png]
